# Effects of light therapy on sleep and circadian rhythm in older type 2 diabetics living in long-term care facilities: a randomized controlled trial

**DOI:** 10.3389/fendo.2024.1307537

**Published:** 2024-02-05

**Authors:** Qin Wang, Shuang Wu, Zhenhua Luo, Lihui Pu, Xiaoxia Wang, Maoting Guo, Mingjiao Zhang, Hongxia Tang, Mengjie Chen, Laixi Kong, Ping Huang, Liyuan Chen, Zhe Li, Dan Zhao, Zhenzhen Xiong

**Affiliations:** ^1^ School of Nursing, Chengdu Medical College, Chengdu, Sichuan, China; ^2^ School of Health and Medicine, Polus International College, Chengdu, Sichuan, China; ^3^ The First Affiliated Hospital of Traditional Chinese Medicine, Chengdu Medical College, Xindu Hospital of Traditional Chinese Medicine, Chengdu, Sichuan, China; ^4^ Menzies Health Institute Queensland & School of Nursing and Midwifery, Griffith University, Brisbane, QLD, Australia; ^5^ Mental Health Center, West China Hospital, Sichuan University, Chengdu, Sichuan, China; ^6^ Sichuan Clinical Medical Research Center for Mental Disorders, Chengdu, Sichuan, China; ^7^ Nursing Key Laboratory of Sichuan Province, Chengdu, Sichuan, China

**Keywords:** older adults, long-term care facility, type 2 diabetes, light therapy, sleep disturbance, circadian rhythm

## Abstract

**Background:**

Light influences the secretion of melatonin in the body and regulates circadian rhythms, which play an important role in sleep and mood. The light level of rooms in long-term care facilities is usually far below the threshold required to regulate the body’s circadian rhythm, and insufficient light can easily lead to sleep and mood disturbances among older residents in nursing homes. Therefore, the objective of this study was to investigate the effects of light therapy on sleep and circadian rhythm in older adults with type 2 diabetes residing in long-term care facilities.

**Methods:**

This study was a prospective, single-blind, randomized controlled trial. Participants were randomly assigned to either the light therapy (LT) group or the control group and received the intervention for four weeks. Primary outcomes included the Pittsburgh Sleep Quality Index (PSQI) and objective sleep parameters recorded by a sleep monitoring bracelet, Morningness-Eveningness Questionnaire (MEQ). The secondary outcome included glycated serum protein (GSP). Data was collected at three time points: at baseline (T0), immediate post-treatment (T1), and 4-week follow-up (T2). A linear mixed model analysis was used to analyzed the data.

**Results:**

We enrolled 45 long-term care residents. Compared with the control group, significant reductions in PSQI scores were observed at T1 and T2. At T2, the sleep score of objective sleep parameters was significantly higher in the LT group compared to the control group. Additionally, compared to the baseline T0, MEQ scores were significantly lower in the LT group at T1 and T2, with no significant difference in the control group. There was no significant difference between groups in glycated serum protein values at T1 and T2. However, compared to T0, glycated serum protein values decreased in the LT group while increased in the control group at T2.

**Conclusion:**

Light therapy had a positive effect on subjective sleep quality and circadian rhythm time type in long-term care residents with type 2 diabetes, and had a possible delayed effect on objective sleep. However, no discernible alterations in blood glucose levels were detected in this study.

## 1 Introduction

The increasing global prevalence of type 2 diabetes, particularly among individuals aged 65 years and older, is a concerning public health issue, the more than 19% of people at least 65 years old have type 2 diabetes worldwide (1). It is estimated that 592 million people around the world will have diabetes by 2035 (2). Sleep disturbance is a global public health problem. It is prevalent in patients with type 2 diabetes. with reported rates of 42-71% in this population (3, 4). Furthermore, long-term care residents are particularly susceptible to sleep disorders, with up to 70% of long-term care residents suffering from sleep problems (5–7), Additionally, a significant proportion of long-term care residents, ranging from 25% to 34%, also suffer from type 2 diabetes (8). Previous research suggests a bidirectional cause-and-effect relationship between type 2 diabetes and sleep disturbance (9–11). Unfortunately, sleep disturbance is an important factor in the deterioration of diabetic patients. It has an impact on the endocrine regulatory system of the body, leading to unstable blood glucose control and aggravating the deterioration of the patient’s condition (12). For example, sleep disturbance can exacerbate the progression of diabetes and diabetic complications by stimulating the body’s sympathetic system, triggering systemic inflammation via the hypothalamic-pituitary-adrenocortical system, thus exhibiting a higher risk of diabetes complications and mortality (13).

Pharmacological treatments such as hypnotic drugs are frequently utilized for sleep disturbance management, however, long-term use may lead to adverse reactions (14), therefore non-pharmacological therapies such as light therapy (LT) are recommended. Light plays an important role in circadian rhythm regulation, with its effects sensed by the intrinsic photosensitive retinal ganglion cells (ipRGC) within the eye and transmitted directly to the suprachiasmatic nucleus (SCN) of the hypothalamus, which regulates sleep-wake rhythms (15). Therefore, exposing patients to bright light therapy is expected to improve their sleep primarily by stabilizing their sleep-wake rhythm. The ipRGC in the eye responds more sensitively to the light of shorter wavelength (blue light) than the light of longer wavelength (red or yellow) (16–19), therefore exposing an individual to blue−enriched light can better regulate sleep-wake circadian rhythms (15, 20), and mitigating sleep disturbances (21–23). The therapeutic efficacy and tolerance of light therapy have been well established, and even for older individuals (24, 25). Given the significance of circadian rhythms for regulating glucose metabolism (26), light therapy may help to improve blood glucose levels in individuals with diabetes.

Light exposure is critical for regulating circadian rhythms, and insufficient exposure to daytime light could be an essential factor contributing to people’s poor sleep outcomes and mood (27, 28). However, light levels in long-term care are usually far below the threshold that regulates people’s circadian rhythms (5, 29, 30). For example, studies have shown that a large percentage of nursing homes have indoor lighting levels lower than 750 lux (31). Even in summer, the median vertical illumination of living rooms in the Norwegian dementia unit was less than 300 lux (32). Furthermore, older adults become less sensitive to light as age increases, which may further reduce the effectiveness of indoor light exposure in stimulating the circadian system (33). In addition, most nursing homes are for older and frail patients whose opportunities for outdoor activities are limited (34), reducing the effective circadian light exposure that older adults receive during the day, which further exacerbating the sleep disorder (33). Insufficient light exposure is related to disrupted sleep, poor sleep quality and depression mood complaints (27, 28). Therefore, light therapy poses a promising non-drug treatment, which may play an important role in treating sleep disorders of type 2 diabetes patients living in long-term care facilities.

Light therapy has been shown to have therapeutic benefits in treating affective disorders such as bipolar disorder, seasonal, and non-seasonal affective disorders (35, 36). Furthermore, light therapy has been demonstrated to has a positive effect on sleep quality, depressive symptoms, and cognitive-behavioral impairment in patients with Parkinson’s and dementia (37–40). Currently, only two studies (41, 42) were identified that examined the effects of light therapy on patients with type 2 diabetes. One study (41) found that while light therapy did not affect insulin sensitivity in type 2 diabetic patients, it did have a positive effect on depressive symptoms in type 2 diabetic patients with high insulin resistance. In another study (42), light therapy was found to improve daytime sleepiness in patients with type 2 diabetes, though no significant improvement was observed in subjective sleep quality. Limited research has been conducted on the use of light therapy as an intervention for diabetic patients, and the available evidence is inconclusive regarding its potential to improve sleep quality and blood glucose levels. Additionally, no studies have been seen to explore the application of light therapy for older type 2 diabetic patients living in long-term care facilities. The aim of this study was to investigate the effect of light therapy on sleep, circadian rhythms, and blood glucose levels in residents with older type 2 diabetes living in long-term care facilities. ​Therefore, we performed a randomized controlled trial, and hypothesized that light therapy (LT) group would have more effects on outcome measures including subjective and objective sleep quality and circadian rhythm, and blood glucose than the control immediately post-treatment and at 4-week follow-up.

## 2 Methods

### 2.1 Trial design

This was a randomized, single-blind trial in which participants were randomly assigned to an intervention (Light therapy) or a control group. Participants were blinded to the grouping. The trial protocol has been approved by the Biomedical Ethics Committee of Chengdu Medical College (Ethical Review Opinion 2021. No. 05). The study was undertaken in accordance with the Declaration of Helsinki. Written informed consent was obtained from each participant before enrollment. Personally identifiable information of participants is anonymously numbered during data analysis to ensure the privacy of participants. The trial was registered at the Chinese Clinical Trial Registry (ChiCTR) with the registration number ChiCTR2200062809. The study is reported according to the Consolidated Standards of Reporting Trials (CONSORT) Trials. (S1 CONSORT Checklist).

### 2.2 Participants and enrollment criteria

Study participants were recruited from three wards of a single long-term care facility (specialized nursing home) in Chengdu, China. The screening of participants was carried out by attending physicians and investigators, and only those individuals who satisfied the following inclusion criteria were enrolled in the study: (1) 65 years or older; (2) diagnosed with type 2 diabetes in accordance with World Health Organization criteria (43) at least 6 months prior to enrollment; (3) diagnosis of any of the circadian rhythm sleep-wake disorders (CRSWD) categories of sleep disorders according to the ICSD-3 (44), and (4) The baseline assessment of the Pittsburgh Sleep Index Scale (PSQI) score is more than 5.

Participants were not enrolled if they (1) have been diagnosed unsuitable for light therapy by an ophthalmologist; (2) have been diagnosed with retinopathy or eye diseases such as cataracts, macular degeneration, glaucoma, or blindness; (3) have ever undergone eye surgery or phototherapy; (4) used photosensitizing drugs in the 30 days before enrollment; (5) currently have acute or severe complications of type 2 diabetes, such as diabetic ketoacidosis, or hyperglycemic hyperosmolar status; (6) had a history of bipolar disorder, severe cognitive impairment or other mental illness diagnosed according to the DSM-5 (45); (7) had a history of cerebral apoplexy, heart failure or other serious physical diseases; (8) Current usage of any medication that affects the circadian rhythms; or (9) being suspected or diagnosed with primary sleep disorders except for insomnia disorder (i.e., restless legs syndrome, sleep-disordered breathing disorder; hypersomnia, or narcolepsy). Participants were not enrolled unless they provided written informed consent.

### 2.3 Sample size

We calculated the minimal sample size of the LT group, N1, and control group, N2, to ensure detection of a significant difference in Pittsburgh Sleep Quality Index (PSQI) score between the intervention and control groups, based on a published study (46). Based on the formula:


$$\text{N}1\;=\text{ N}2\;=\;2\;{\left[\;\left({\text{t}}_{\alpha /2}+t_{\beta /2}\right)\text{S}／\text{δ}\right]}^{2},$$

where δ = 9.79 - 6.81 = 2.98 and S=2.79 (46) and a standard normal distribution table (47) indicated t_α/2_ = 1.96 and t_β/2_ = 1.282, we calculated N1 = N2 = 18.39. We increased these values to 22 subjects in each group in order to compensate for 20% loss to follow-up.

### 2.4 Randomization, concealment and blinding

Participants were randomly divided into LT group or control group using online random number generator software (www.random.org; Dublin, Ireland). The intervener grouped the participants according to sequence numbers randomly generated by the computer-generated list. The randomly generated list order was hidden before the intervention assignment. Participants themselves were blinded to their group assignment. The light therapy group uses bright 1500 Lux light therapy glasses and the control group uses a virtual low-light light therapy glasses model. Despite the difference in nature in the bright light and virtual low light conditions, the shape of the glasses devices worn by the two groups was similar and the older adults had less autonomous activity, which the participants were not aware of it. To avoid interference between the two groups, participants housed in the same room at the nursing room facility were assigned to the same group. The researchers and caregivers kept information about the light therapy and control groups strictly confidential, and participants were not aware of the differences between the two types of light, and participants did not know whether they are receiving the actual light therapy or the virtual low-light condition.

### 2.5 The intervention

The study was conducted in three phases for nine weeks. During the baseline period of seven days, data was collected on objective and subjective sleep parameters, glycosylated serum protein, and depressive symptoms. During the intervention phase of four weeks, participants in the LT group wore Luminette light therapy glasses (Lucimed, Villers-Le-Bouillet, Belgium) with a corneal level of 1500 lux (blue-enriched white light at a wavelength of 468 nm), while the control group wore custom-made light therapy glasses identical in appearance to those of the LT group but emitting a corneal level of 0.3 lux (faint yellow light), which did not affect circadian rhythms (48). Luminette glasses can deliver the same light therapy as conventional light boxes operating at 10,000 lux (49, 50). Both groups will wear their glasses every morning from 9:00 to 10:00 am. After the intervention, participants were followed up for four weeks, during which the same assessments were carried out as during the baseline phase.

The researcher conducted regular assessments to verify the adherence of participants to wearing light therapy glasses throughout the intervention, and maintain a comprehensive record of their compliance. Additionally, the nursing staff actively monitor participants’ adherence to wearing the glasses during their daily routines, thereby ensuring continuous compliance with the prescribed light therapy protocol.

### 2.6 Assessment

Trained assessors collected data from participants at three time points: at baseline (T0), immediate post-treatment (T1), and 4-week follow-up (T2). Primary outcomes included Pittsburgh Sleep Quality Index (PSQI) score, objective sleep parameters recorded by a sleep monitoring bracelet, Morningness-Eveningness Questionnaire (MEQ) score, and secondary outcome included glycated serum protein (GSP) value.

#### 2.6.1 Subjective sleep quality

At T0, T1 and T2, participants completed the Chinese version (51) of the Pittsburgh Sleep Quality Index (PSQI) (52) to assess their nocturnal sleep quality and daytime sleepiness during the preceding four weeks. The scale comprises seven dimensions: subjective sleep quality, sleep latency, sleep duration, sleep efficiency, sleep disorders, use of sleep medications, and daytime dysfunction. The subscore on each dimension can range from 0 to 3, so the total score can range from 0 to 21, with higher total score indicating worse sleep quality. An overall score > 5 is be defined as a sleeping disorder (53). Cronbach’s α for the Chinese version of the PSQI is 0.84, and retest reliability is 0.81 (51).

#### 2.6.2 Objective sleep

At baseline, throughout weeks 4 of the intervention and throughout weeks 4 of follow-up (7 days), participants wore a sleep monitor bracelet (Honor Band 5i, Huawei, Shenzhen, China) daily to record objective sleep parameters. The sleep monitoring bracelet is a wristband smart wearable device that features cardiopulmonary coupling (CPC) technology, which can accurately analyze sleep patterns (54, 55). The sleep monitor bracelet automatically record daily data on sleeping time and waking time, total sleep time (TST), light sleep duration (LST), deep sleep duration (DST), rapid eye movement (REM) sleep, and time of awakening (TOA). The device determines an overall “sleep quality score”. The total score can range from 0 to 100, with higher score indicating better sleep quality.

#### 2.6.3 Circadian rhythms

At T0, T1 and T2, participants completed the Chinese version (56) of the Morningness-eveningness Questionnaire (MEQ) (57). The scale is a chronotype classification tool for natural trends of circadian rhythm to assess the type of circadian rhythm. In this study, MEQ scores were compared to determine the trend change of circadian rhythm. The scale contains 19 items, and individuals scoring 16-30 will be classified as “absolute night type”; 31-41, “moderate night type”; 42-58, “intermediate type”; 59-69, “moderate morning type”; and 70-86, “absolute morning type”. Cronbach’s α for the Chinese MEQ is 0.701-0.738, and retest reliability is 0.638-0.831 (56). In this study, MEQ scores will be compared among three time points to determine the change trend of circadian rhythm.

#### 2.6.4 Blood glucose level

At T0, T1, T2, peripheral venous blood (2-3 mL) were collected from subjects at 9:00 am and assayed for levels of glycosylated serum protein (GSP) as an index of mean glycemic control (58) using the nitroblue tetrazolium method on a Cobas8000 automatic biochemical analyzer (Roche, Germany).

### 2.7 Statistical analysis

Data was analyzed using SPSS 26.0 (IBM, Chicago, IL, USA). For intention-to-treat (ITT) analysis, the last observation carried forward (LOCF) method were used to impute any missing data of the objective questionnaire and glycated serum protein data (59). When poor compliance in wearing the sleep monitoring bracelet results in less than <5 days of recorded sleep parameters, the sleep parameter data was discarded. Measurement data conforming to normal distribution were expressed as mean ± standard deviation, differences between the two groups were assessed by Student’s *t* test; skewed continuous data by M (Q1, Q3), and differences between were measured by non-parametric rank-sum test. Counting data were described by *n* (%), and the significance of differences between groups was assessed by Chi-square test. Differences between different time points within each group were assessed for significance using linear mixed models (60). Group (intervention *vs*. control), time (treated as categorical with levels at T0, T1 and T2), and the group-by-time interaction were included as fixed effects in the model. Bonferroni correction was used for *post hoc* analysis. Differences associated with *p* < 0.05 were considered significant.

## 3 Results

### 3.1 Baseline patient characteristics

**Figure 1:**
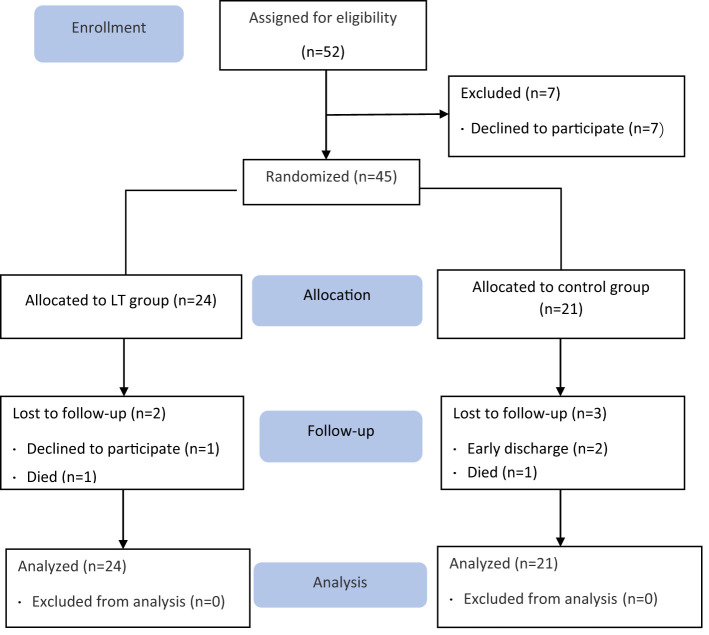
CONSOR flow diagram.

**Figure 2:**
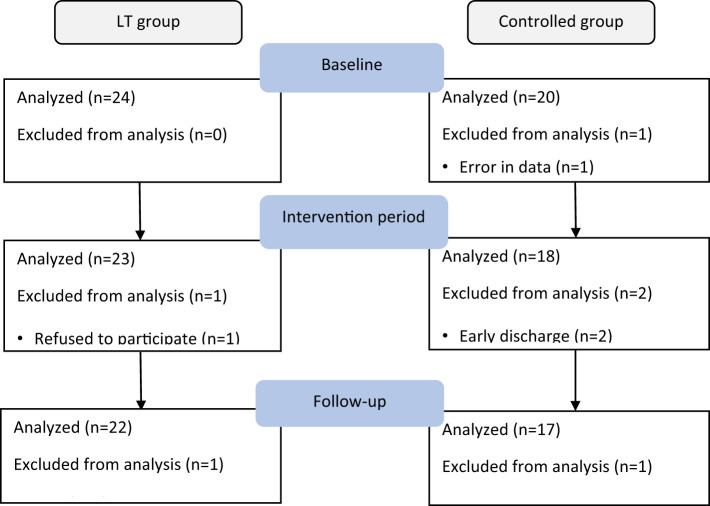
Summarizes the number of participants included in each analysis for sleep parameters as measured by sleep monitoring bracelet at each data collection time point and the reasons for missing data.

A total of 45 older adults with type 2 diabetes and sleep disturbances in nursing homes were included in this study (see the flow chart in **Figure 1**), with a mean age of 85.33 ± 5.924 (years), 16 cases were (35.56%) males and 29 cases were (64.44%) females. There were 24 cases (53.3%) in the LT group and 21 cases (46.7%) in the control group. There were no significant differences between the two groups in PSQI, MEQ scores, sleep parameters measured by sleep monitoring bracelet and GSP values. **Table 1** gives additional demographic aspects of the subjects, including length of long-term care residence, duration of type 2 diabetes, duration of sleep disturbance, co-morbidity, type of dementia diagnosis and sleep medication use, as well as the baseline of outcome measures. **Figure 2** shows the number of participants included in each analysis or sleep parameters at each time point and the reasons for missing data.

**Table 1:** Descriptive statistics at baseline for the 45 participants.

	Control group (n=21)	LT group (n=24)	*P*
Age (mean, SD)	86.33 (5.58)	84.46 (6.19)	0.295
Sex, n (%)			
Women	12 (57.1)	17 (70.8)	0.338
Man	9 (42.9)	7 (29.2)	
Duration of type 2 diabetes since diagnosis, years, mean (SD)	14.83 (10.92)	16.13 (9.67)	0.676
Duration of sleep disturbance, years, M (Q1, Q3)	3.00 (5.50,1.50)	2.00 (3.75,1.00)	0.267
Residence time of long-term care settings, years, mean (SD)	2.59 (1.70)	2.88 (1.88)	0.599
Number of comorbid chronic diseases, n (%)			
2	1 (4.8)	4 (16.7)	0.205
≥3	20 (95.2)	20 (83.3)	
Dementia diagnoses, n (%)			
No diagnosis	14 (66.7)	10 (41.7)	0.096
AD	4 (19.0)	12 (50.0)	
VD	3 (14.3)	2 (8.3)	
Types of sleeping drugs, n (%)			
None	7(33.3)	9 (37.5)	0.567
Alprazolam	6 (28.6)	5 (20.8)	
Estazolam	7 (33.3)	6 (25.0)	
Clonazepam	1 (4.8)	4 (16.7)	
PSQI score (mean, SD)	15.10 (0.58)	16.13 (0.54)	0.138
Sleep parameter (mean, SD)			
Sleep duration	485.45 (35.97)	534.31 (33.32)	0.241
LST, min	355.55 (30.95)	379.29 (28.63)	0.414
DST, min	85.00 (12.11)	97.80 (11.17)	0.406
REM, min	41.00 (6.00)	57.82 (5.56)	0.061
TOA, min	3.60 (0.30)	2.81 (0.28)	0.085
Daytime sleep time, min	160.25 (17.02)	163.31 (15.80)	0.870
Sleep quality score	64.80 (2.00)	68.54 (1.84)	0.234
MEQ (mean, SD)	66.47 (0.80)	66.76 (0.85)	0.766
GSP (mean, SD)	272.52 (10.30)	278.47 (9.53)	0.850

AD, Alzheimer’s Disease; VD, Vascular Dementia; PSQI, Pittsburgh Sleep Quality Index; TST, Total Sleep Time; LST, Light Sleep Time; DST, Deep Sleep Time; REM, Rapid Eye Movement, TOA, Times of Awakening; MEQ, Morningness-Eveningness Questionnaire; GSP, glycated serum protein.

### 3.2 Outcomes

**Figure 3:**
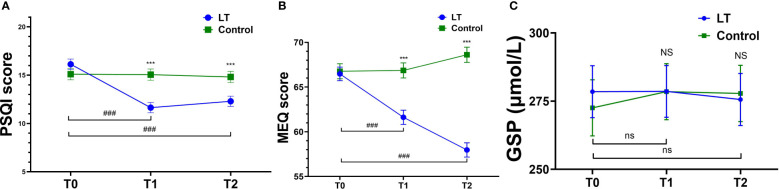
Comparison of PSQ, MEQ and GSP mean scores at T0, T1 and T2. Pittsburgh Sleep Quality Index (PSQI) **(A)**, Morningness-Eveningness Questionnaire (MEQ) **(B)**, Glycosylated Serum Protein (GSP) **(C)**; T0, baseline; T1, immediate post-treatment; 4-week follow-up (T2). ***p < 0.001 (*vs*. the control group); ^###^ *p* < 0.001 (*vs*. T0 in LT group); NS (*p* > 0.05, *vs*. control group); ns (*p* > 0.05, *vs*. T0 in LT group).

The linear mixed model was used to analyze the data, and the fixed effects results showed that the differences in group effect, time effect, and time × group interaction effect of PSQI and MEQ scores of patients in the two groups at different times were statistically significant (*p* < 0.05), and The difference in GSP was not statistically significant (*p* > 0.05), indicating that the magnitude of change in PSQI and MEQ scores was significantly different between the two groups before and after the intervention, and there was no significant change in GSP in both groups before and after the intervention (see **Figure 3** and **Table 2**). Furthermore, the linear mixed model was used to analyze the objective sleep parameters recorded by the sleep monitoring bracelet, and the fixed-effects results showed that the time effects of TST and LST were statistically different between the two groups (*p* < 0.05). The group effect, time effect, and time × group interaction effect of other sleep parameters were not statistically different between the two groups of participants (*p* > 0.05) (see **Table 2**).

**Table 2:** Comparison of outcomes between the light therapy (LT) group and control group (mean ± standard deviation).

N=45	Group	T0	T1	T2	Time effect	Group effect	Time × Group interaction
					*p value* ^b^		
PSQI^a^	LT	16.13 ± 0.54	11.63 ± 0.54	12.30 ± 0.54	**<0.001**	**0.021**	**<0.001**
	Control	15.10 ± 0.58	15.05 ± 0.58	14.81 ± 0.58			
MEQ^a^	LT	66.47 ± 0.76	61.62 ± 0.80	57.97 ± 0.80	**<0.001**	**<0.001**	**<0.001**
	Control	66.76 ± 0.85	66.86 ± 0.85	68.62 ± 0.85			
GSP^a^	LT	278.47 ± 9.53	278.56 ± 9.50	275.59 ± 9.53	0.798	0.919	0.802
	Control	272.52 ± 10.30	278.48 ± 10.30	277.81 ± 10.30			
TST, min	LT	534.31 ± 33.32	590.71 ± 33.32	567.76 ± 34.00	**0.010**	0.153	0.455
	Control	485.45 ± 35.97	558.10 ± 38.10	473.13 ± 39.57			
LST, min	LT	379.29 ± 28.63	420.81 ± 28.63	406.53 ± 29.18	**0.007**	0.065	0.676
	Control	355.55 ± 30.95	434.88 ± 32.59	388.87 ± 33.84			
DST, min	LT	97.80 ± 11.17	112.11 ± 11.17	108.291 ± 11.36	0.111	0.281	0.903
	Control	85.00 ± 12.11	97.47 ± 12.63	88.18 ± 13.09			
REM sleep	LT	57.82 ± 5.56	50.52 ± 5.56	50.62 ± 5.67	0.463	0.163	0.103
	Control	41.00 ± 6.00	49.80 ± 6.35	39.48 ± 6.60			
TOA, mim	LT	2.81 ± 0.28	3.14 ± 0.28	2.98 ± 0.29	0.717	0.455	0.170
	Control	3.60 ± 0.30	3.08 ± 0.33	2.97 ± 0.34			
Daytime sleep time, min	LT	163.31 ± 15.80	139.71 ± 15.80	129.89 ± 16.12	0.576	0.196	0.204
	Control	160.25 ± 17.02	179.93 ± 18.12	165.75 ± 18.81			
Sleep score	LT	68.54 ± 1.84	70.05 ± 1.84	69.97 ± 1.87	0.591	**0.055**	0.708
	Control	64.80 ± 2.00	65.10 ± 2.07	64.06 ± 2.14			

a Intention-to-treat analysis population.

b By Linear mixed model.

PSQI, Pittsburgh Sleep Quality Index; MEQ, Morningness-Eveningness Questionnaire; GSP, Glycated Serum Protein; TST, Total Sleep Time; LST, Light Sleep Time; DST, Deep Sleep Time; REM, Rapid Eye Movement, TOA, Times of Awakening. Bold values indicate that the difference was statistically significant or close to statistical significance.

#### 3.2.1 Sensitivity analysis

We performed a sensitivity analysis. A total of 40 (out of 45) subjects were included in the analysis (18 in the LT group and 22 in the control group). There were no significant differences in the baseline characteristics of the two groups. The results of sensitivity analysis showed statistically significant differences in the group effect, time effect, and time × group interaction effect for PSQI and MEQ scores across time in both groups (*p* < 0.05), consistent with the ITT results, indicating the stability of the statistical results (see **Table 3**).

**Table 3:** Sensitivity analysis.

N=40^a^		T0	T1	T2	Time effect	Group effect	Time × Group interaction
					*p value^b^*		
PSQI	LT	16.100 ± 0.57	11.39 ± 0.57	12.10 ± 0.57	<0.001*	0.013*	<0.001*
	Control	15.03 ± 0.63	15.04 ± 0.62	14.86 ± 0.63			
MEQ	LT	66.47 ± 0.80	61.62 ± 0.80	57.97 ± 0.80	<0.001*	<0.001*	<0.001*
	Control	66.76 ± 0.85	66.86 ± 0.85	68.62 ± 0.85			
GSP	LT	276.55 ± 9.42	282.77 ± 10.28	282.60 ± 10.35	0.689	0.742	0.682
	Control	273.44 ± 10.35	277.04 ± 9.393	273.42 ± 9.43			

Comparison of outcomes between the light therapy (LT) group and control group (mean ± standard deviation).

a Per-protocol analysis population.

b By Linear mixed model.

PSQI, Pittsburgh Sleep Quality Index; MEQ, Morningness-Eveningness Questionnaire; GSP, Glycosylated Serum Protein.

* Statistically significant difference.

#### 3.2.2 Post hot analysis

Regarding subjective sleep, the results showed that the PSQI scores of the LT group were significantly lower than those of the control group at T1 (β = -3.413, 95% CI: -4.988 to -1.839, *p* < 0.001) and T2 (β = -2.512, 95% CI: -4.088 to -0.936, *p* = 0.002) (see **Table 4**).

**Table 4:** Results of the *post hoc* analyses.

Outcomes		Changes between time points						Group difference					
		△T0 to T1			△T0 to T2			T1			T2		
		β [SE]	95% Cl	*p* Value^b^	β [SE]	95% Cl	*p* Value^b^	β [SE]	95% Cl	*p* Value^b^	β [SE]	95% Cl	*p* Value^b^
PSQI^a^	LT	-4.497 [0.468]	[-5.642,-3.351]	**<0.001**	-3.833 [0.488]	[-5.054,-2.613]	**<0.001**	-3.413 [0.790]	[-4.988,-1.839]	**<0.001**	-2.512 [0.791]	[-4.088,-0.936]	**0.002**
	Control	-0.048 [0.495]	[-1.260,1.164]	1.000	-0.286 [0.521]	[-1.590,1.019]	1.000						
MEQ^a^	LT	-4.852 [1.110]	[-7.566,-2.137]	**<0.001**	-8.500 [1.126]	[-11.232,-5.768]	**<0.001**	-5.242 [1.164]	[-7.545,-2.939]	**<0.001**	-10.652 [1.164]	[-12.955,-8.349]	**<0.001**
	Control	0.095 [1.185]	[-2.807,2.998]	1.000	1.857 [1.204]	[-1.064,4.778]	0.376						
**TST, min**	LT	72.645 [36.066]	[-15.627,160.917]	0.142	-12.324 [45.315]	[-122.573,97.924]	1.000	32.615 [50.611]	[-67.97,133.19]	0.521	94.637 [52.165]	[-9.023,198.297]	**0.073**
	Control	56.401 [31.924]	[-21.767,134.568]	0.244	33.454 [39.944]	[-63.762,130.670]	1.000						
**LST, min**	LT	79.331 [28.860]	[8.763,149.900]	**0.022**	33.319 [36.984]	[-56.680,123.318]	1.000	-14.067 [43.380]	[-100.315,72.181]	0.747	17.657 [44.685]	[-71.168,106.482]	0.694
	Control	41.525 [25.519]	[-20.896,103.947]	0.323	27.238 [32.536]	[-51.967,106.442]	1.000						
**Sleep score**	LT	0.302 [1.47]	[-3.300,3.904]	1.000	-0.738 [1.972]	[-5.548,4.071]	1.000	4.945 [2.774]	[-0.594,10.485]	0.079	5.903 [2.844]	[0.228,11.578]	**0.042**
	Control	1.511 [1.297]	[-1.667,4.690]	0.744	1.429 [1.726]	[-2.781,5.639]	1.000						

*Post hoc* analysis of time effect, group effect, Time × Group interaction effect p<0.05 in outcome indicators. The group effect of sleep score p=0.055 was performed *post hoc* analysis.

PSQI, Pittsburgh Sleep Quality Index; MEQ, Morningness-Eveningness Questionnaire; TST, Total Sleep Time; LST, Light Sleep Time.

a Intention-to-treat analysis population.

b By Linear mixed model.

Bold values indicate that the difference was statistically significant or close to statistical significance.

The linear mixed model analysis of sleep parameters measured by the sleep monitoring bracelet showed no statistical differences between two groups at T1 (*p* > 0.05). However, at T2, the LT group had a significantly higher sleep score than the control group (β = 5.903, 95% CI: 0.228 to 11.578, *p* = 0.042) (see **Table 4**).

Regarding the type of circadian rhythm, the results showed that compared with T0, MEQ scores were significantly lower in the LT group at T1 (β = -4.852, 95% CI: -7.566 to -2.137, *p* < 0.001) and T2 (β = -8.500, 95% CI: 11.232 to -5.768, *p* < 0.001), with no significant difference in the control group (*p* > 0.05) (see **Table 4**). The type of circadian rhythm in the LT group changed from “intermediate wakefulness” (66.47 ± 0.80 points) to “intermediate” (57.97 ± 0.80 points) at T2.

## 4 Discussion

In this prospective, single-blind, randomized controlled trial, we investigated the effects of light therapy on sleep disturbances, circadian rhythm and blood glucose in older adults with type 2 diabetes with sleep disturbance living in long-term care facilities. This study was conducted in long-term care facilities, and the included older adults with diabetes were all aged 70 and above. Therefore, the results of this study reflect the situation of older adults with diabetes in long-term care facilities in China.

In the present study, we found that light therapy improved the subjective sleep quality of patients. Our findings indicate that light therapy may have a direct and lasting effect on subjective sleep quality in people with type 2 diabetes. This is consistent with the results of Figueiro et al. (40) who showed a significant reduction in overall PSQI score in Alzheimer’s patients in nursing facilities after light intervention. Results also showed that PSQI scores remained lower four weeks after the light intervention, indicating that the effects of light therapy on subjective sleep quality were long-lasting.

The results of sleep parameters measured by the sleep bracelet showed a significant improvement in sleep scores at T2 in the LT group and an increase in total nighttime sleep time compared to the control group after 4 weeks of follow-up, but not significant (p = 0.073). The results of this study were similar to the findings of Kim et al. (39) who found no significant changes in objective sleep parameters after the intervention, but there was a trend towards an improvement in total nighttime sleep duration in the intervention group compared to the control group, suggesting that light therapy may have a delayed effect on objective sleep quality. Longer periods of circadian stimulation may be required to determine the effectiveness of light on patients to consolidate sleep (61–63). However, previous studies have shown the benefits of light therapy on objective sleep parameters assessed by sleep monitors. A previous study by Sloane et al. (64) found that the use of high-intensity ambient light significantly increased the duration of nighttime sleep in patients with dementia. Figueiro et al. (65) found improvement in TST and SE as measured by activity loggers in most patients with dementia using a blue-white light device.

In this study, we investigated the effect of light therapy on sleep quality in older type 2 diabetics living in long-term care facilities. This study is innovative in terms of population selection. To our knowledge, this is the first study of light therapy in older type 2 diabetics living in long-term care facilities. The results showed that light therapy showed significant effects in improving patients’ subjective sleep quality, although in terms of objective sleep parameters, light therapy only produced significant improvements in sleep scores. However, sleep quality itself is a more subjective feeling, and we observed a significant improvement in patients’ subjective perception of sleep quality. This improvement helped to reduce anxiety and depression in patients and promote psychological well-being, which could play an important role in preventing further disease progression in patients with type 2 diabetes.

No significant changes in objective sleep parameters were found in this study. A possible explanation for this finding is the short duration of the intervention. In a study with a prolonged (3.5 years) lighting intervention (66), objective nighttime sleep duration was significantly increased and the improvement in sleep quality was consistently greater in the test group than the control condition. Thus, the small or insignificant changes in objective sleep parameters in our study may be related to the duration of light exposure, as four weeks may be relatively short, and our findings need to be validated by further studies with longer intervention durations.

The type of circadian rhythm in the LT group changed from “intermediate wakefulness” (66.762 ± 0.850 minutes) at T0 to “intermediate” (57.967 ± 0.795 points) at T2, which prolonged the patients’ morning waking time, suggesting that light therapy can appropriately adjust the circadian rhythm and can stabilize nighttime sleep. Similarly, Baandrup et al. (67) found that a light intervention improved circadian rhythms and circadian activity patterns in older adults with cognitive impairment living in nursing homes. Figueiro et al. (65) also found that a tailored lighting intervention had a modulating effect on circadian rhythms in patients with dementia. This observed phenomenon is posited to arise from the regulatory effects of light therapy on sleep-wake cycles through the modulation of intrinsically photosensitive retinal ganglion cells (ipRGC), with direct delivery to the suprachiasmatic nucleus (SCN) of the hypothalamus, thereby contributing to an enhancement in sleep quality (20). Inadequate levels of indoor light in nursing homes may not be conducive to proper entrainment of circadian rhythms. Moreover, sleep disturbances in older patients with type 2 diabetes can result in altered sleep structure, characterized by increased awakenings, earlier awakenings, and greater difficulty in falling back asleep after awakening (68). The reason for the results of this study may be that light regulates circadian rhythms, solidifies nighttime sleep, and prolongs wakefulness in type 2 diabetic patients.

Although the results of this study did not find significant differences in glycated serum protein values between the two groups, several studies have shown a significant association between sleep disturbances and circadian rhythm disturbances and increased glycemic level control in type 2 diabetes (69, 70). Sleep disturbances and circadian rhythm disturbances impair β-cell function and insulin sensitivity, leading to impaired glucose tolerance, which severely affects glycemic level control and thus exacerbates the progression of type 2 diabetes (71). It has also been shown that light improves sleep structure in patients with type 2 diabetes. Therefore, improving sleep quality through light therapy is important for glycemic control in type 2 diabetes (42). Future studies could select patients with poorer levels of glycemic control for longer interventions to better explore the effects of light therapy on the level of glycemic control in patients with type 2 diabetes.

This study was conducted in a single-center study with limited sample size and may lack the representativeness of the sample. Future randomized, controlled, multicenter studies can be undertaken to further confirm the effectiveness of light therapy on sleep quality and glycemic control in older patients with type 2 diabetes. Furthermore, circadian biochemical indicators were not collected in this study to objectively assess the circadian phase of patients, such as dark-light melatonin onset levels. Melatonin serves as a major marker of circadian rhythm changes. Future studies could be designed to collect dark-light melatonin episodes levels as an objective assessment of circadian rhythm changes. Besides, the diabetes complications and the use of anti-diabetic medications should be considered in future studies to provide more robust evidence for the benefits of light therapy for people with type 2 diabetes. Moreover, an evaluation of sleep behavior and associated sleep parameters at approximately 15 years prior to their diabetes diagnosis, should also be considered due to the impact of sleep habits on outcomes.

## 5 Conclusion

In conclusion, our findings provide evidence for a positive effect of light therapy on subjective sleep among older adults with type 2 diabetes and sleep disturbance in long-term care facilities, contributing to the regulation of circadian rhythm time type, with a possible delayed effect on objective sleep. No changes in blood glucose levels were found in this study for the time being, and it may be necessary that longer periods of light exposure in patients with poorer levels of glycemic control would help to detect the beneficial effects of light therapy.

## Data availability statement

The original contributions presented in the study are included in the article/supplementary material. Further inquiries can be directed to the corresponding authors.

## Ethics statement

The studies involving humans were approved by Biomedical Ethics Committee of Chengdu Medical College (Ethical Review Opinion 2021. No. 05). The studies were conducted in accordance with the local legislation and institutional requirements. Written informed consent for participation in this study was provided by the participants’ legal guardians/next of kin.

## Author contributions

QW: Conceptualization, Data curation, Formal analysis, Investigation, Methodology, Software, Validation, Writing – original draft, Writing – review & editing. SW: Formal analysis, Writing – review & editing. ZHL: Formal analysis, Validation, Writing – review & editing. LP: Conceptualization, Methodology, Supervision, Validation, Writing – review & editing. XW: Data curation, Investigation, Writing – review & editing. MG: Investigation, Methodology, Writing – review & editing. MZ: Data curation, Writing – review & editing. HT: Formal Analysis, Writing – review & editing. MC: Formal analysis, Writing – review & editing. PH: Writing – review & editing. LK: Writing – review & editing, Formal analysis. LC: Writing – review & editing. ZL: Conceptualization, Methodology, Supervision, Validation, Writing – review & editing. DZ: Conceptualization, Supervision, Validation, Writing – review & editing. ZX: Investigation, Resources, Supervision, Validation, Writing – original draft, Writing – review & editing.
